# Renal Function in Children with Cyanotic Congenital Heart Disease: Pre- and Post-Cardiac Surgery Evaluation

**Published:** 2014-01-25

**Authors:** Hamid Amoozgar, Mitra Basiratnia, Fatemeh Ghasemi

**Affiliations:** 1 Department of Pediatric Cardiology, Nemazee Hospital; 2 Shiraz Nephrology and Urology Research Center; 3 Department of Pediatrics, Nemazee Hospital, Shiraz University of Medical Sciences, Shiraz, Iran

**Keywords:** Congenital Heart Disease, Nephropathy, Cardiac Surgery, Cyanosis, Microalbuminuria

## Abstract

***Objective:*** Cyanotic congenital heart diseases (CCHDs) are a series of cardiac anomalies that have long been recognized as a potential cause of nephropathy. There have been few reports on renal impairment in patients with CCHD before and after corrective cardiac surgery. The aim of this study was to evaluate the prevalence of renal dysfunction before and after cardiac surgery and the impact of some risk factors on final renal outcome.

***Methods:*** Thirty children with CCHD who had done corrective cardiac surgery in the previous 6 months were enrolled in this study. All data prior to surgery were collected from the charts. Post-operation data including blood and spot urine samples were taken simultaneously for CBC, Cr, and uric acid and 24 hour urine was collected for microalbumin and Cr during the follow up visits. Pre- and post-operation parameters were compared to study the impact of cardiac surgery on renal function.

***Findings*** ***:*** Pre- and post-operative GFRs were not significantly different. Final GFR was significantly and inversely associated with pre- and post-operation age (*P*=0.008 *r*=-0.48, P=0.03 *r*=-0.38). Three (10%) patients had microalbuminuria. The prevalence of microalbuminuria in children older than 10 years was 30%. There was no link between microalbuminuria and age, GFR, and hematocrit (*P*=0.1, *P*=0.3, *P*=0.3, respectively). Patients with preoperation hematocrit >45 had a significantly lower final GFR compared to children with HCT <45 (83.7±6.5 vs 111.10.2, *P*=0.001). The mean uric acid fraction (FEua) excretion was 8.21±4.75. Pre-operative HCT was inversely associated to FEua (*P*=0.01, *r*=-0.44). There was no relationship between FEua and age, serum uric acid, and GFR (*P*=0.7, *P*=0.4, *P*=0.2).

***Conclusion:*** Children with CCHD are at increased risk of renal injury which is related more to the duration of cyanosis and higher degree of hematocrit level. To lower the risk, corrective cardiac surgery is recommended to be done as soon as possible to improve renal function and stop more renal impairment.

## Introduction

Congenital cyanotic heart diseases (CCHDs) are a series of cardiac anomalies that can induce severe impairment of various organs including kidneys, respiration, vascular bed, hemostasis, red blood mass, central nervous system, digits, and long bones^[^^1^^,^^2^^]^. Several studies have revealed that nephropathy is a prominent feature and a potential complication of CCHD^[^^3^^-^^6^^]^. Disorders of renal function in CCHD take the form of abnormal glomerular and tubular function. Regarding glomerular dysfunction, decreased GFR and macro or microalbuminuria have been reported^[^^7^^]^. 

**Fig. 1:**
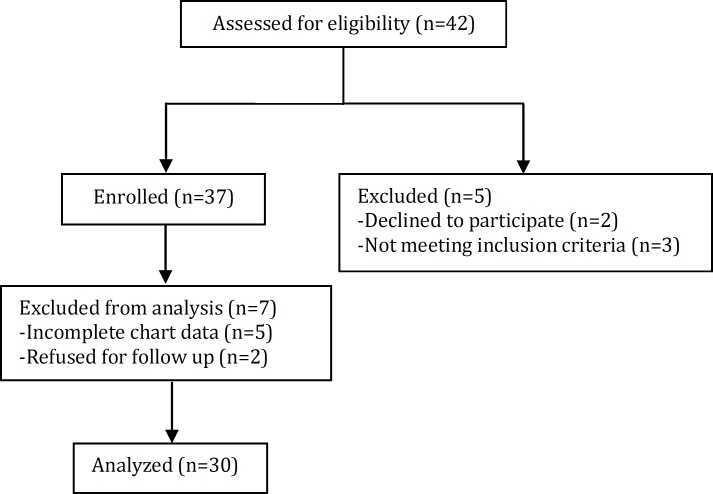
Flow chart of participants in the study

The hallmarks of glomerular changes in CCHD are glomerulomegaly, capillary dilatation, mesangial cell proliferation, and glomerulosclerosis^[^^8^^]^. The structural integrity and function of proximal tubules have also been studied, indicating tubular dysfunction and loss of its integrity in patients with CCHD^[^^9^^]^. 

 Duration of cyanosis and an elevated hematocrit are suspected to be involved as the major risk factors for the development of cyanotic nephropathy in patients with CCHD^[^^10^^]^.

 Most studies have investigated the risk factors for nephropathy in patients with CCHD, but few have analyzed the extent of nephropathy after corrective cardiac surgery and its comparison with preoperative values^[^^11^^]^. The aim of the present study was to evaluate the prevalence of renal impairment in patients with CCHD before and after cardiac surgery, and to clarify the impact of several risk factors for nephropathy on the post-operative course.

## Subjects and Methods

In a cross-sectional study over a period of 8 months (September 2011- April 2012), 30 patients with CCHD who referred to the Pediatric Cardiology Clinic affiliated with Shiraz University of Medical Sciences, and at least 6 months had passed from their corrective cardiac surgery were selected (Fig 1). The study was reviewed and approved by the university review board and ethics committee. All parents have given informed consent prior to the study. Exclusion criteria were renal anomalies, endocarditis, diabetes mellitus, acute infection, and recently used nephrotoxic drugs. Pre-operation clinical and laboratory data including age, height, CBC, and serum Cr were recruited from admission chart at the time of surgery. Post-operation data including blood and spot urine samples were taken simultaneously for CBC, Cr, and uric acid during the last follow up visit (at least 6 months after cardiac surgery). Uric acid was measured in plasma by spectrophotometry method and plasma and urine cretinine by Jaffe kinetic method. FEUA as a tubular marker was calculated by formula: (urine UA×plasma Cr)/(plasma UA×urine Cr). 24 hour urine was collected for micro-albumin and Cr. Microalbumin assay was done by enzyme linked immunosorbent. Estimated GFR was calculated by Schwartz formula [GFR (ml/min/1.73m2)= κ×height (cm)/serum Cr (mg/dl)]. 

 Statistical analysis was performed using SPSS, version 16. The parametric variables were presented as mean±SD and analyzed by paired t-test and Pearson correlation test as appropriate. Chi-square was used for non-parametric variables. *P*<0.05 was considered statistically significant.

**Fig. 2A, B:**
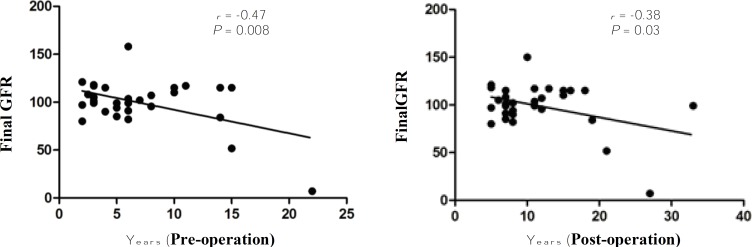
Scatter plot of the association between pre- and post-operation age and final GFR among children with CCHD

## Findings

The study population comprised 30 patients with CCHD with a mean age of 11.47±6.7 years. The mean age at the time of surgery was 7.16±4.93 years and the mean duration of follow up after cardiac surgery was 5.3±1 years. Fifteen patients were diagnosed as Tetralogy of Fallot and underwent total correction, 8 had pulmonary atresia and ventricular septal defect, in whom total correction with homograph was done. The remaining 7 patients who had double outlet right ventricle and pulmonary stenosis had also underwent total correction with homograph.

 There was a significant decrease in hematocrit after cardiac surgery (*P*=0.001, *r*=0.58). Pre- and post-operative GFRs were not significantly different (91.97±16.76 vs 99.39±25.10, *P*=0.2). Pre-operation GFRs were as follows: 41% had GFR>90, 53% 60-90, and 6% <60%. After 

corrective surgery, these figures changed to: GFR>90 in 76%, 60-90 in 18%, and <60 in 6% of the patients. Final GFR was significantly and inversely associated with pre- and post-operation age (*P*=0.008 *r*=-0.48, *P*=0.03 *r*=-0.38) (Fig. 2A, B). Three (10%) patients had microalbuminuria. The prevalence of microalbuminuria in children older than 10 years was 30%. There was no link between microalbuminuria and age, GFR, and hematocrit (*P*=0.12, *P*=0.3, *P*=0.28, respectively). 

**Fig. 3:**
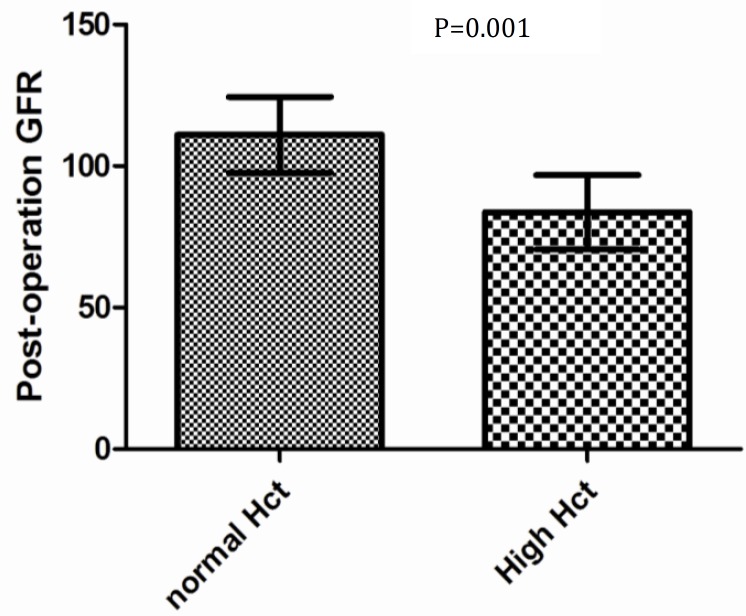
Box plot of the association between hematocrit and final GFR

 Patients with pre-operation hematocrit >45 had a significantly lower final GFR compared to children with HCT <45 (83.7±6.5 vs 111.10.2, *P*=0.001) (Fig. 3). The mean uric acid fraction (FEua) excretion was 8.21±4.75. 

Pre-operative HCT was inversely associated to FEua (*P*=0.01 *r*=-0.44). There was no relationship between FEua and age, serum uric acid, and GFR (*P*=0.77, *P*=0.4, *P*=0.2).

## Discussion

It has been well-recognized that cyanotic congenital heart disease can lead to functional and morphological renal derangements in adult patients^[^^2^^]^. The present study showed that renal dysfunction can also be evident during early childhood and aggravates with longer duration of cyanosis and higher degrees of erythrocytosis, and can be prevented by earlier corrective cardiac surgery.

 Our study revealed that more than half of the patients had GFR lower than 90cc/min/1.73m^2^. Although there was no significant difference between pre- and post-operative GFR, about one third of the patients with mild renal dysfunction showed an increase in GFR (GFR>90) after cardiac surgery. It seems that reduction of GFR prior to operation is related more to the hemodynamic changes in glomerulus rather than structural derangements. In agreement with the result of our study, Awad et al showed a significant improvement in glomerular and tubular functions after palliative cardiac surgery in children with CCHD^[^^11^^]^. Severe hypoxia and secondary erythropoiesis can affect renal function through failure of maximum compensatory hyperfiltration to overcome the reduction of renal plasma flow. It has been shown previously that reduction in hematocrit is accompanied by an increase in renal blood and plasma flow, and a decrease in efferent glomerular arteriolar resistance^[^^12^^,^^13^^]^. 

 Our study revealed that older children had more renal impairment in comparison with younger patients. Therefore, the present study is supported by other reports indicating that duration of cyanosis is one of the most important predisposing factors for renal dysfunction^[^^10^^,^^14^^]^. In Martinez et al.’s study, the majority of the adult patients had GFR lower than 90 cc/min/1.73m^2^, but the proportion with GFR lower than 60 was higher than that of the present study (23% vs 6%)^[^^14^^]^. Dittrich et al identified that the duration of cyanosis is one of the major risk factors for the development of glomerulopathy, and the risk rises sharply during the second decade of life in patients with CCHD^[^^10^^]^. Our results were in accordance with the mentioned study in that 86% (6 out of 7) of children with more than ten years CCHD, had GFR 60-90 and 14% had GFR less than 15^[^^1^^]^.

 In addition to hemodynamic changes, longstanding cyanosis can proceed to structural damage including tubules and glomerules. One of the early markers of glomerular damage is microalbuminuria. There is no data related to preoperative microalbuminuria, but 10% (3 out of 30) of our patients had microalbuminuria after surgery and all of them were more than 10 years old. No association between microalbuminuria and duration of cyanosis was found, which might be due to the younger age of our patients (mean age 7.16±4.93 years) in comparison to other reports^[^^15^^,^^16^^]^. Krull et al reported proteinuria in 44% of patients with CCHD mostly in the second decade of life, and only one of nine children under 10 years of age had microalbuminuria^[^^15^^]^. Akita et al assessed 16 patients with CCHD and documented proteinuria and albuminuria in the six oldest patients (aged 15-28 years)^[^^16^^]^. Zheng et al investigated glomerular and tubular damage in 58 children ≤3 years and despite showing higher normalized microalbumin in children with CCHD in comparison to the control group, only one of the children with severe cyanosis had glomerular injury^[^^17^^]^. These studies along with the present study reiterate that the microalbuminuria as a marker of glomerular injury does not commonly occur in early stage in children with CCHD; therefore, patients with CCHD might be followed by microalbuminuria, at least after the second decade of life. 

 What is the cause of microalbuminuria? There are several hypotheses. First, the erythrocytosis of CCHD is accompanied by intraglomerular shear stress and release of nitric oxide (NO), which leads to dilated, engorged, and enlarged glomeruli with selective dilatation of afferent arterioles that increase intraglomerular pressure and alteration of permeability of the glomerular basement membrane and consequent proteinuria^[^^18^^-^^20^^]^. Second, the reduction of peritubular blood flow secondary to hyperviscosity might be responsible for the increase in intra-glomerular pressure and the resulting proteinuria^[^^21^^]^. Third, podocyte stress induced by increase in glomerular capillary surface area results in podocyte hypertrophy and consequent dysfunction^[^^22^^]^.

 We found that children with higher hematocrit had a significantly lower post-operative GFR than children with lower hematocrit. Although we had no tissue diagnosis to determine the cause of reduction of GFR, Perloff et al have documented that high viscosity can induce endothelial shear stress and resulting NO release, subsequent glomerular enlargement, mesangial cell proliferation and matrix expansion, and subsequent hyalinized glomeruli, atrophic tubules, and interstitial fibrosis^[^^18^^]^. Howenstine et al showed that noncardiac polycythemia such as polycythemia vera and erythrocytosis resulting from intrinsic lung disease can lead to glomerular lesions similar if not identical to the previously mentioned histology^[^^23^^]^.

 One limitation of the present study was the lack of data on O2 saturation to be able to detect the impact of hypoxia on GFR, but previous studies have confirmed that polycythemia rather than hypoxia is a risk factor for cyanotic nephropathy^[^^11^^,^^24^^]^.

 According to several studies, tubular injury occurs in the early stage of CCHD^[^^9^^,^^17^^]^.

 Zheng et al showed that tubular injury can occur in very early course of CCHD, even before glomerular damage^[^^17^^]^. Agras et al detected increased urinary tubular markers in a study population aged 0-13 years within the first decade of life^[^^9^^]^.

 Urinary N-acetyl-β-D-glucosaminidase (NAG) and α1-microglobulin (α1-MG) are commonly used for evaluation of early tubular dysfunction. We could not measure these biomarkers due to our financial limitations; instead, we calculated fraction excretion of uric acid as a less accurate marker of tubular injury. The mean fraction excretion of uric acid was relatively low (8.21± 4.75) and it was, inversely, more closely related to hematocrit rather than to the age, GFR, and serum uric acid. This result is in accordance with the report of Awad et al who demonstrated that hematocrit was significantly correlated with urinary parameters of tubular damage including NAG and α1microglobulin. He also demonstrated that acute changes in renal hemodynamics are the result of acute changes in hematocrit induced by palliative cardiac surgery and can lead to significant improvement of functional and structural tubular integrity.

## Conclusion

Children with CCHD are at increased risk of renal injury which is related more to the duration of cyanosis and higher degrees of hematocrit level. To lower the risk, corrective cardiac surgery should be done as soon as possible to improve the renal function and prevent further renal impairment.
